# Veratrum Viride in Fevers

**Published:** 1857-03

**Authors:** A. W. Dewey

**Affiliations:** Strawtown, Ind.


ARTICLE IV.—*Veratrum Viride in Fevers.* By A. W. DEWEY, M.D. Strawtown, Ind.

PROF. DAVIS:

*Dear Sir*—I hope I shall not intrude by again reporting a case or two in my practice of the last season, any portion whereof you are at liberty to publish or omit. My chief object is to add my testimony in favor of the use of Veratrum Viride, which I have this season for the first made use of in practice. I was not a little distrustful of the expediency of adopting even a much needed remedy, the powers of which I had never tested. At present I feel no hesitation, rather a pleasure, in adding to the testimony in favor of its use; believing it to be one of the most powerful, if not the most reliable, agent for controlling arterial excitement of which our Materia Medica can boast.

On the introduction of a new remedy, we see, from the earliest records of the profession, a constant tendency in those who advocate its adoption to overrate its powers and to magnify anticipated benefits. Desirous to avoid that common error, we shall not pronounce unequivocally for its use in all cases of increased arterial action, but will patiently wait till further experience has more fully demonstrated the extent and applicability of its powers. And allow me also to indulge the hope that those whose greater experience and better abilities, with wider fields for observation, furnish greater facilities for determining, will turn their attention to it, and report their conclusions to the profession. In my limited experience with it, I have not yet discovered its diaphoretic action, as spoken of by Dr. Mills, in the *Journal* for January, 1855. I did not, however, use it to the extent of producing emesis, nor in combination with spts. of nitre.

*Case I.*—Miss M. M. aged nineteen, for some five or six days had suffered from common intermittent, so they supposed —herself and friends—and had been endeavoring to treat it themselves by cathartics and quinine. On the 21st of July, the fever was more intense, and came on without a chill or any well-marked cold stage. Continued all night, with very slight remission next morning, followed by exacerbation. By noon on the 22d it was decided to summon medical aid. I visited her at 1 P.M. Found the system in a high state of febrile action; face flushed; surface dry and hot; pulse one hundred and twelve to one hundred and fifteen; severe pain in head, back, and limbs; considerable tenderness in the epigastrium; great thirst; tongue rather red and moderately coated—bowels having been rather freely acted upon by some patent pills kept in the house.

I gave two grains calomel and five of Doveri, and in a few minutes gave five drops of the saturated tinc. of veratrum viride, in a little sweetened water. Ordered four drops of the tincture to be given every hour, till the fever abated; also, in four hours, another calomel powder, combined as before. Some camphorated tincture of opium was left to restrain the action of the pills on the bowels, one tea-spoonful of which was given shortly afterward.

Called on 23d at 9 A.M. Found her more comfortable than she had been for two days; some headache still, and the tenderness of the stomach only partially relieved. Four doses of the tinc. had been taken. Pulse, seventy-six; unable to sit up on account of vertigo. Ordered the ver. vird. three drops every hour till 2 P.M.; then, if there was no fever, quinine and Doveri every two hours until 8. Should the fever rise, a calomel powder was to be given, and the tinc. continued through the day. There was no fever. Three pills of blue mass were given at 9 in evening, and quinine every three hours till noon of the 24th. Called at 1 P.M. Said she was well; pulse seventy; surface moist; tongue still coated. Ordered blue pill again at night, with quinine and muriate of sodium next day, for three doses at intervals of three hours. Promised no further visits. Saw her a few days subsequently—had no return of fever.

The main feature in this case, as well as a few other easily-managed ones that followed, is the omission of all other remedies, on the second day of the treatment, until after the hour of the expected return of fever—which failed in its appearance; but as our fevers were unusually mild last season, I scarcely know how much credit is due to the article.

The following was more marked and satisfactory:—

*Case II.*—Mrs. L. R. aged twenty-seven, of sanguino-nervous temperament, near seven months *enceinte*, was taken, July 26th, with dysentery. Called in a Dr. P. next-door neighbor, who claimed to have read a regular course in the wonderful system of Homœopathy, and to possess, in common with his preceptor and Homœopathic brethren, an extraordinary ability for the successful treatment of all diseases flesh is heir to, but more especially in the treatment of diseases of the bowels. He attended his patient very closely. All agreed that her symptoms were more favorable on Monday, 28th. On the 29th, the disease had stolen a march upon him—discharges were more frequent and bloody; tenesmus worse than at any time previously, and more febrile action. His exertions and unremitting attention, from the history afterward given, really deserved better success—but the gods were inauspicious. The disease progresses apace; a more violent fever, with parching thirst, supervenes; tenesmus and tormina severe beyond measure; prostration complete during the night. The next morning (30th) her husband is summoned from his labor, some miles distant; arrives in the afternoon; finds his redoubtable Doctor in great tribulation, and the life of his companion in imminent peril; orders a messenger for me in haste. He had, by request, consented to wait and give me a history of the case and treatment; but got in a very great hurry and left ten minutes before my arrival: thus I was deprived of one very important item in this communication, and his subsequent removal to some part of Iowa has put it out of my power to supply the deplorable deficit. But to the patient, reluctantly taken charge of:—The countenance was haggard, and expressive of great suffering; surface hot, dry, and husky; pulse one hundred and thirty-five to one hundred and forty, thread-like; tongue dry, and coated with brown fur, with red edges and tip, the body of which was tremulous; extreme prostration; voice scarcely audible when endeavoring to speak; tenderness so great, that the least attempt at pressure on any part of the abdomen caused her to start and cry out; with lower extremities flexed: in short every appearance of violent entero peritonitis. My prognosis was certainly very unfavorable, and I would have decided upon following in the footsteps of my illustrious predecessor, by unceremoniously decamping, had not my sympathy for the patient predominated even over my regard for the learned son of Hahnemann.

I first gave gum opii three grains, camphor about the same, with half grain ipecac. In five minutes, gave seven drops of tinc. ver. vird in some elm water that was at hand, and ordered fomentation of hops, hot as could be borne, to the abdomen. I should have stated, that intermitting pains had come on some two hours before, with pain in the back, which led to the opinion that premature delivery might be looked for. The venerable ladies present asserted that such was inevitable—they knew about such things—had seen 'Mrs. So and So, who died, was in just such a way.' Well, in fact, I was more than half inclined to agree with them. Sat down to note the effects of the medicine, and to hear divers apologies for being called secondly to wait upon the case. The uterine pains not subsiding in three quarters of an hour, I gave three grains more opium and camph. and in fifteen minutes six drops more of the ver. vird. In ten minutes more there was a marked improvement: pulse down to one hundred and fifteen; pains subsiding; the breathing more full and free. Remembering, with satisfaction, the good effects of sulp. acid in cases analogous, I put up elix. vit. ʒij.; sulp. morp. 2grs.; aromatic syrup rhei, ℥j.; and filled the 2oz. vial with elm water. Ordered a tea-spoonful of this every two hours, and four drops ver. vird. every two hours, alternating; the latter to be discontinued after three doses more were given.

Called next morning at 9—patient improved beyond expectation. No medicine had been given for the last three hours, waiting my arrival and allowing the patient to sleep. Pulse eighty per minute. Convalescence progressed regularly to complete recovery in a few days, with the usual treatment in such cases. Ten weeks subsequently delivered her of a healthy male child—both did well.

